# Dr. George D. Slocum

**Published:** 1903-03

**Authors:** 


Dr. George D. Slocum, of Marine City, Mich., U. of B. '61, died recently at his home, aged 62 years.

